# The Grievance Dictionary: Understanding threatening language use

**DOI:** 10.3758/s13428-021-01536-2

**Published:** 2021-03-23

**Authors:** Isabelle van der Vegt, Maximilian Mozes, Bennett Kleinberg, Paul Gill

**Affiliations:** 1 Department of Security and Crime Science, University College London, 35 Tavistock Square, London, WC1H 9EZ UK; 2 Dawes Centre for Future Crime, University College London, London, UK; 3 Department of Computer Science, University College London, London, UK; 4 Department of Methodology and Statistics, Tilburg University, Tilburg, Netherlands

**Keywords:** Psycholinguistic dictionary, Threat assessment, Grievances, Violence, Language, LIWC

## Abstract

This paper introduces the Grievance Dictionary, a psycholinguistic dictionary that can be used to automatically understand language use in the context of grievance-fueled violence threat assessment. We describe the development of the dictionary, which was informed by suggestions from experienced threat assessment practitioners. These suggestions and subsequent human and computational word list generation resulted in a dictionary of 20,502 words annotated by 2318 participants. The dictionary was validated by applying it to texts written by violent and non-violent individuals, showing strong evidence for a difference between populations in several dictionary categories. Further classification tasks showed promising performance, but future improvements are still needed. Finally, we provide instructions and suggestions for the use of the Grievance Dictionary by security professionals and (violence) researchers.

## Introduction

Psycholinguistic dictionaries assume language use reflects the emotions and cognitive processes of a text author (Pennebaker, Boyd, Jordan, & Blackburn, 2015; Pennebaker & King, 1999). Consequently, these processes are thought to be measurable, for example by examining a text for words that refer to a specific process or concept. One of the most prominent examples of a word-count based psycholinguistic dictionary is the Linguistic Inquiry and Word Count (LIWC; Pennebaker et al., 2015). It provides a “method for studying the various emotional, cognitive, and structural components present in individuals’ verbal and written speech samples” (Pennebaker et al., 2015, p.1). In short, LIWC seeks to measure variables relating to linguistic style (e.g., word count, pronouns, number of verbs), psychological processes (e.g., anxiety, power), and personal concerns (e.g., family, religion). Other dictionaries (e.g., Wmatrix, Rayson, 2008; Empath, Fast, Chen, & Bernstein, 2016; Moral Foundations Dictionary, Frimer, Boghrati, Haidt, Graham, & Dehgani, 2019; IBM Watson Tone Analyzer^1^) also exist and measure different concepts and categories, however LIWC remains pre-eminent within research circles.

Such psycholinguistic dictionaries are increasingly used to understand and detect extreme, threatening, or hateful language on the web (e.g., Davidson, Warmsley, Macy, & Weber, 2017; Kleinberg, van der Vegt, & Gill, 2020; Scrivens, Davies, & Frank, 2018). They are also used to inform automatic linguistic threat assessment (e.g., Akrami et al., 2018). Threat assessment can cover a range of threats of violence including violent extremism, public mass murder, school shootings, and targeted violence against public figures. These forms of violence share a similar genesis, typically involve some form of pre-planning, and are driven by a grievance (Corner et al., 2018). They are also often signaled ahead of time in some form of written communication (Gill, 2020). Typically, research on automatic linguistic threat assessment tries to discriminate between texts authored by perpetrators of grievance-fueled violence from some form of non-violent control or comparison group (Baele, 2017; Kaati, Shrestha, & Cohen, 2016).

However, the psycholinguistic dictionaries frequently used in these contexts are met with two important limitations. Firstly, standard psycholinguistic dictionaries have not been developed for the purpose of assessing grievance-fueled language and therefore do not measure constructs that may be of interest to researchers and threat assessment practitioners. Although the LIWC provides categories such as anxiety and anger, we propose that key concepts for threat assessment and violence research are absent in this and other dictionaries. As a result, previous work on grievance-fueled violence that used the LIWC (e.g., Baele, 2017; Kaati, Shrestha, & Cohen, 2016) may not have been specific enough in terms of the linguistic measures used to indicate potential violence. Second, the content and construction procedure of existing dictionaries is often unclear because descriptions of how and why certain words have been selected are scarce. Yet, it is vital to be transparent about the development of these because of the far-reaching consequences of false positives and negatives within the context of threat assessment. In the UK, the ALGO-CARE framework suggests that algorithms used in the context of policing need to be explainable, in that decision-making rules and the impact of each factor on the outcome is available (Oswald, Grace, Urwin, & Barnes, 2018). In short, it is highly important for practitioners and researchers to understand the capabilities and limitations of a given dictionary. For many available dictionaries and threat assessment software^2^, the contents of wordlists or other ‘under the hood’ operations are not available to its users, and thus cannot be adequately evaluated or explained. This possibility is desirable and necessary if such systems are to be used in practice.

To address the aforementioned limitations, this paper presents the Grievance Dictionary, which specifically aims to measure psychological and social concepts that are of interest in the context of grievance-fueled violence-threat assessment. First, the Grievance Dictionary is specifically aimed at measuring concepts that are of interest in threat assessment and violence research and practice. Its aim is to supplement measures obtained through dictionaries such as the LIWC with concepts that are specifically relevant to the threat assessment domain. Second, the Grievance Dictionary is transparent in terms of its construction and final format. All data collected are made available freely (e.g., for researchers and practitioners), including the words that are included in the final dictionary as well as background characteristics of consulted experts. Third, the dictionary is not restricted to a specific type of violence or extremism. Any threat, abuse, or violent writing fueled by a grievance can be assessed with the Grievance Dictionary. This would apply to a wide spectrum of phenomena, including right- and left-wing extremism, religious extremism, and (in many cases) threats directed at public officials. Resultingly, dictionary terms will not necessarily need to be continuously updated as is the case for other domain-specific dictionaries.

In the following section, we discuss psycholinguistic dictionaries and their use in threat assessment. In part one, we discuss how the Grievance Dictionary was developed through expert consultation, human and computational word list generation, and crowdsourced annotations. We also perform a psychometric evaluation for each dictionary category. In part two, we present empirical results using the final dictionary. The dictionary is validated by performing statistical comparisons as well as classification tasks on several datasets. We conclude with a general discussion of the dictionary development and validation, as well as possible future avenues.

## Automatic linguistic threat assessment and grievance-fueled violence

In the automatic linguistic approach to grievance-fueled violence, particular attention has been paid to the writings of terrorists and (online) extremists (Baele, 2017; Kaati, Shrestha, & Cohen, 2016; Kop, Read, & Walker, 2019). A few studies examined lone-actor terrorist manifestos for various psycholinguistic variables using the LIWC (Baele, 2017; Kaati, Shrestha, & Cohen, 2016). These studies compared lone-actor terrorist writings to the writings of several different populations, such as non-violent activists (e.g., Martin Luther King, Nelson Mandela), standard control writings and emotional writings (i.e., ‘baseline’ texts expressing low and high emotionality, respectively), and personal blogs (Kaati, Shrestha, & Cohen, 2016). In several studies, lone-actor terrorist manifestos differed from control texts on several LIWC variables. For example, they contained higher proportions of negative emotion words including anger (Baele, 2017; Kaati, Shrestha, & Cohen, 2016), lower levels of positive emotion and friendship words (Kaati, Shrestha, & Cohen, 2016), and more power-related words (Baele, 2017; Kaati, Shrestha, & Cohen, 2016). Similar research focused on ‘incel’ (i.e., involuntary celibate) forums. Jaki et al. (2019) compared 50,000 messages from an incel forum to 50,000 neutral ‘control’ texts extracted from Wikipedia articles and random English tweets via LIWC software. Incel messages contained more swear words and negative emotion, such as anger and anxiety.

Besides the use of the LIWC, several studies in extremism additionally use custom-made ‘expert dictionaries’. For these dictionaries, domain experts are consulted to develop wordlists that cover the terms used by a specific population. For example, Smith, Wakeford, Cribbin, Barnett, and Hou (2020) developed an expert dictionary for ISIS vernacular after consulting ‘terrorism and extremism experts from government and the security and defense sectors’ (Smith et al., 2020, p.6). Figea, Kaati, and Scrivens (2016) developed one for racism, aggression, and worries on a white supremacy forum.

Using the LIWC as well as expert dictionaries, other studies go beyond statistical comparisons alone, and classify violent from non-violent texts via machine learning. In a study of a white supremacy forum, all 73 LIWC categories and three expert dictionaries relating to worries, racism and aggression were used as features (Figea et al., 2016). LIWC categories for religion (e.g., ‘Muslim’, ‘church’), see (e.g., ‘view’, ‘saw’) and third-person pronouns (e.g., ‘they’, ‘them’) proved to be important linguistic characteristics for classifying racism posts. The LIWC categories for anger (e.g., ‘hate’, ‘kill’) and an expert dictionary category for aggression were important for recognizing both worries and aggression in the posts, achieving accuracy rates between 80 and 93%. In another effort, classification tasks using the LIWC output as predictors distinguished between lone-actor terrorist manifestos, texts written by non-violent activists, texts from personal blogs, forum postings on Stormfront (a white supremacy forum), and personal interest forum postings (Kaati, Shrestha, & Sardella, 2016). In one of the tasks where the aim was to distinguish between terrorist texts and Stormfront posts, LIWC categories relating to negative emotion (e.g., ‘sad’, ‘angry’), time (e.g., ‘before’, ‘often), and seeing (e.g., ‘appear’, ‘show’) were important features for classification with an accuracy of 90%.

In earlier work, the concepts of hate and violence were measured on American and Middle Eastern dark web forums (Abbasi & Chen, 2007). The authors utilized a custom dictionary containing words and phrases from the forums related to violence and hate (the content of the dictionary was not made available). The results indicated that Middle Eastern forums scored higher than American forums in terms of violence. Forums from both regions did not differ in terms of hate. Similarly, Chen (2008) proposed an automated method for analyzing affect within two jihadist dark web forums. Up to 909,039 messages were collected from the forums, of which 500 were utilized to manually construct a dictionary for violence, anger, hate, and racism. One of the forums, known to be more radical, was indeed found to contain higher levels of violence, anger, hate, and racism than the other (Chen, 2008).

Custom dictionaries created through expert consultation also potentially suffer from a third limitation in addition to the two noted in the introduction. They are often highly domain-dependent and non-transparent regarding the population of experts consulted. By consulting domain experts (e.g., in right-wing extremism, radical Islam), the dictionaries are specifically attuned to a specific type of violence or extremism. The nature of online communication in these populations is that language is community-specific and constantly changes (Farrell, Araque, Fernandez, & Alani, 2020; Shrestha, Kaati, & Cohen, 2017). Some fringe communities may also continuously adapt their language use to evade content moderation filters on social media platforms which automatically delete or flag posts with specific word use (van der Vegt, Gill, et al., 2019). As a result of these phenomena, dictionaries would have to be continuously updated to capture the appropriate jargon. Furthermore, custom expert dictionaries are referenced in Abbasi and Chen (2007), Chen (2008), Figea et al. (2016) and Smith et al. (2020), but little is said about what the consultation process entailed and why those consulted can be considered experts. In short, readers are expected to trust the judgment of the researchers and experts without having access to the specifications of the tool.

### Transparency statement

The approach to developing the Grievance Dictionary was fully pre-registered before data collection: https://osf.io/szvm7. All data and materials are available on the Open Science Framework: https://osf.io/3grd6/. A user guide for the dictionary can be found there too.

## The Grievance Dictionary

### Part I: Dictionary development

The dictionary development consisted of five phases. (1) Threat assessment experts suggested dictionary categories. (2) Human subjects generated seed terms for each category. (3) Computational linguistics methods augmented the word list. (4) Human annotators rated word candidates on their fit into a set of categories. (5) The internal reliability for each dictionary category was assessed and their correlation with LIWC2015 categories was computed.

#### Phase 1: Expert survey

An online survey was sent out to experts within the field of threat assessment. Participants were professional contacts of the involved researchers in the field of threat assessment and terrorism research. Participants were asked the following:

Imagine you are tasked with assessing whether a piece of text signals a threat to commit violence against a designated area, individual, or entity. It may be a physical letter or an online message that you are asked to examine. In short, you are trying to judge whether the person who wrote the text will act on their threat. What do you look for in the text to assess its threat level? Please mention all relevant factors that come to mind.

The response to this question was an open text box, with no word limit. Following this, participants could add any other relevant factors that came to mind (again with an open answer response) and were asked about their professional experience in threat assessment (in years) and with linguistic threat assessment in particular (on a 10-point scale, 1 = no experience, 10 = a lot of experience).

In total, 21 responses were gathered. On average the participants had 16 years of experience with threat assessment (SD = 8.84, range, 2–30 years). Overall, the participants indicated they had significant experience with threat assessment based on language, with a mean score of 8.17 (SD = 2.04, on a scale from 1–10).

Based on the survey responses gathered, it became clear that assessing the threat of violence through language relies on a wide variety of factors. In order to adequately measure these factors, they need to be condensed into psycholinguistic categories (similar to the LIWC). The lead author categorized free text responses. For example, the concepts ‘preparation’, ‘rehearsal’, ‘developing capacity’, ‘refining method’, or ‘developing opportunity’, were all coded as a single category relating to ‘planning’. In total, this resulted in 79 categories (available on the OSF). The categories could broadly be defined to relate to the content of a communication (e.g., direct threat, violence, relationship), emotional processes (e.g., anger, frustration, desperation), mental health aspects (e.g., psychosis, delusional jealousy, paranoia), the communication style (e.g., unusual grammar, politeness, incoherence), and meta-linguistic factors (e.g., number of communications, font, use of graphics). Lastly, the lead author selected categories that could feasibly be represented as a psycholinguistic wordlist, serving as an overarching category (e.g., including ‘weaponry’ but excluding ‘mentioning target’ because it is too situation-specific). This resulted in a final selection of 22 categories (Table 1).

**Table 1:** Dictionary categories with example words (defined in later steps)

Category	Examples
Planning	long-term, tactic, organize
Violence	bloodshed, fight, bullet
Weaponry	AK-47, ammo, fire arm
Help seeking	support, SOS, save
Hate	enemy, loathe, hatred
Frustration	annoyed, problem, powerless
Suicide	die, overdose, last resort
Threat	warn, danger, unsafe
Grievance	wrong, disappointed, injustice
Fixation	obsess, possess, watch
Desperation	sorrow, last chance, urgent
Deadline	time run out, due date, upcoming
Murder	kill, stab, fatal
Relationship	marry, romantic, love
Loneliness	disconnected, nobody, abandon
Surveillance	spy, CCTV, monitor
Soldier	fighter, battle, patriot
Honor	integrity, hero, brave
Impostor	impersonate, fraudulent, undercover
Jealousy	cheat, resent, bitter
God	pray, holy, almighty
Paranoia	suspicious, conspiracy, suspect

#### Phase 2: Seed word generation

Human subjects generated seed words for each category from Phase 1. A total of 13 participants suggested words for the categories in an online survey. Participants were all PhD students at English-speaking universities (full details of the sample are reported in the supplementary materials on OSF). For each category, participants were asked to write down all the words that came to mind, considering the category as an over-arching concept for the words they noted down. This resulted in a total of 1951 seed words across categories. Instructions for the word generation task as well as the resulting words for each category are available in the online materials.

#### Phase 3: Word list extension

Two processes extended the word list. First, WordNet (Fellbaum, 1998) provided semantic associations for each seed word. This tool provides a lexical database of English words, grouped into ‘cognitive synonyms’ of meaningfully related words, which are added to the wordlists (e.g., ‘knife’ is supplemented with ‘dagger’, ‘machete’, and ‘shiv’). All words related to the initial seed words were added to the list of the respective category.

Second, we obtained pre-trained word embeddings for each candidate word using GloVe, an unsupervised learning approach trained on a 6 billion-word corpus (Pennington, Socher, & Manning, 2014). GloVe represents words as a vector in multi-dimensional space (embeddings) which aim to encode semantic relationships between individual words based on the contexts in which they appear. This means that words which are similar in meaning have vector representations that are close to each other (based on a similarity measure) in the resulting vector space (e.g., a word embedding for ‘gun’ appears close to ‘handgun’, ‘pistol’, ‘firearm’, etc. in the learned vector space). For the dictionary, each seed word across all categories was supplemented with its ten nearest neighbor words in terms of cosine similarity. After removing duplicates obtained through WordNet and the embeddings, the final resulting wordlist across all categories contained 24,322 words. These words may appear in multiple categories (e.g., ‘knife’ may appear in both the weaponry and murder category).

#### Phase 4a: Word list rating

Human annotators rated all 24,322 words obtained through Phase 3 for the extent to which they fit within their respective category. An online task was developed where participants were presented with a category, a word, and the option to select, on a scale, ‘how well the word displayed fits into the above category’ (0 = does not fit at all, 10 = fits perfectly). They also had the option to select ‘I do not know this word’. After reading instructions and consenting to participating, a total of 100 words (i.e., a random sample of 100 word-category pairs, with words shown for their associated category only) were rated by each participant. Participants were recruited through the crowdsourcing platform Prolific Academic and remunerated for their time. Human workers were only eligible to participate if their first language was English. Interspersed between normal items, four attention checks were included (e.g., ‘This is an attention check. Rate this word with 9 to continue’).

In sum, the 24,322 words of the extended wordlist were rated by 2318 online participants. A total of 238,366 ratings were obtained, with each word receiving at least seven ratings, with an average of 9.42 ratings per word. All ratings from participants who failed at least one of the attention checks were removed (1.81%). Words for which the majority (50% or more) of participants indicated that they did not know the word, were also removed from the dictionary (0.39%). Following this, all dictionary words were stemmed and the ratings averaged per word stem (e.g., the ratings for ‘friendship’, ‘friendly’, and ‘friends’ were combined into a single score for the stem ‘friend’). This resulted in a final list of 20,502 words, each of which could appear in more than one category.

#### Phase 4b: Scoring methods

Departing from the rated word list, several versions of the Grievance Dictionary can be used. First, it is important to note that in all versions the words in the dictionary are stemmed (e.g., ‘friendship’ and ‘friends’ are equated to the word stem ‘friend’) in order to find more possible matches. Word stemming is done with Porter’s stemming algorithm (Porter, 2001) using the quanteda *R* package (Benoit et al., 2018).

Three approaches to using the dictionary are discussed. The first two rely on proportional scoring, based on word counts. Following the LIWC, we may wish to only retain words which received a high rating for belonging to a specific category (Pennebaker et al., 2015). In this first version, we would retain only those words which received an average rating of 7 or higher, resulting in a dictionary with 3643 words. This version is used for evaluation and validation in this paper. An alternative second version retains words with a score of 5 or higher, resulting in a dictionary with 7588 words. In both of these versions, scoring the texts follows the same approach as the LIWC, which is based on word count. When the dictionary is applied to a text, the incoming text is first stemmed and lowercased using *quanteda* (Benoit et al., 2018) in the same way that has been done with the words in the Grievance Dictionary. The number of words in the texts are subsequently counted, and a warning is given if the word count is below 25 (with the option to remove texts that fall under this threshold). This procedure derives from the evaluation of the LIWC2015, which only included texts with a minimum word count of 25, and further instructions that results are more ‘trustworthy’ when the word count is higher^3^. We expect the same holds for the Grievance Dictionary. Therefore, we similarly recommend using the Grievance Dictionary on texts with 25 words or more.

Following this, each word in the dictionary is searched in the respective text and a document-feature matrix (i.e., the rows represent a document and the columns represent individual features/dictionary categories) is returned, based on which we can calculate the proportion of a text that belongs to each dictionary category (i.e., frequency of all word matches in category / all words in text) using *quanteda*. As an alternative to measuring proportions per category (22 features), documents could also be represented as a function of all words (3643 or 7588 features) in these versions of the Grievance Dictionary.

The third approach relies on average scoring, using the ratings assigned to each word through crowdsourcing. This version of the dictionary makes use of all 20,502 words and their associated average goodness-of-fit rating, assigning each word match in a text the appropriate weight. To measure each category for a text of interest, the average weight of all word matches per category is reported^4^. While the first version using proportional scoring of words with a mean score of 7 and higher is used in this paper, alternative versions are available on the Open Science Framework.

#### Phase 5: Psychometric dictionary evaluation

To assess the quality of the dictionary, it is important to examine the internal consistency of each category by measuring whether the words in each category yield a similar score for the respective category. We compute Cronbach’s alpha using the proportional occurrence of each word in the 22 categories for a total of 17,583 texts across four corpora (Table 2). Similar to the development of LIWC2015, we use a varied selection of texts to compute reliability, including texts from deception detection experiments (Kleinberg, van der Vegt, Arntz, & Verschuere, 2019), novels (Lahiri, 2014), movie reviews (Maas et al., 2011), and Reddit posts (Demszky et al., 2020).

**Table 2:** Corpora used for internal consistency computation

Corpus	Number of documents (number of tokens)
Deception detection experiments^a^	2547 (454,217)
Novels (Lahiri, 2014)	3036 (247,142,420)
IMDB movie reviews (Maas et al., 2011)	50,000 (13,934,687)
Reddit posts (Demszky et al., 2020)	70,000 (1,081,539)

*Note.* ^a^Hotel reviews (Ott, Choi, Cardie, & Hancock, 2011; Ott, Cardie, & Hancock, 2013), descriptions of past and planned activities (Kleinberg et al., 2019)

When assessing the internal reliability of psychological tests, typically a Cronbach’s alpha score of 0.70 or higher is considered acceptable (Taber, 2018). Cronbach’s alpha ranges between 0 to 1 and is based on the number of items and the correlation between them, where a score of 1 represents perfect inter-item correlation, such that the items adequately measure the same underlying concept. When computing internal consistency for wordlists, each word serves as an ‘item’ for the measurement of the overarching category. The proportional occurrence of each word in the 22 categories is thus computed for each of the four corpora, in order to compute the correlation between words in a category (i.e., the Cronbach’s alpha score for the category). We report the average Cronbach’s alpha across the four corpora for each category.

As raised in Pennebaker et al. (2015), assessing the reliability of dictionaries is somewhat more complicated. In language, similar concepts are typically not repeated several times; once something has been said it is generally not necessary to be said again. In contrast, similar concepts may be assessed repeatedly in psychological test items. Thus, it has been argued that an acceptable alpha score for dictionary categories will be lower than that for a psychological test (Pennebaker et al., 2015).

A psychometric evaluation was performed for each version of the dictionary (words with a rating of 7 or higher, words with a rating of 5 or higher, weighted words). The results reported from here onwards concern the dictionary using words with a rating of 7 or higher, because this dictionary performed best (results for the other versions are available on the OSF). The average alpha scores across corpora are reported in Table 3. The highest reliability of 0.37 is achieved for the category ‘soldier’, followed by 0.36 for ‘violence’. The lowest scores (0.12, 0.16) were found for the categories ‘fixation’ and ‘grievance’, respectively, which possibly shows that these concepts are difficult to reliably measure with the current approach. The average reliability achieved across categories was 0.26 (SD = 0.07). This average reliability is somewhat close to the average reliability of 0.34 achieved with the LIWC 2015. The alpha scores for the LIWC2015 ranged between 0.04 and 0.69, whereas ours range between 0.12 and 0.37.

**Table 3:** Internal consistency scores

Category	Cronbach’s alpha
Deadline	0.27
Desperation	0.21
Fixation	0.12
Frustration	0.22
God	0.35
Grievance	0.16
Hate	0.30
Help	0.19
Honor	0.26
Impostor	0.19
Jealousy	0.21
Loneliness	0.18
Murder	0.35
Paranoia	0.23
Planning	0.31
Relationship	0.33
Soldier	0.37
Suicide	0.26
Surveillance	0.25
Threat	0.30
Violence	0.36
Weaponry	0.34

In addition to internal reliability, we also assessed whether and how the Grievance Dictionary categories correlated with existing LIWC categories. We correlated Grievance Dictionary scores with LIWC scores (using document-feature-matrices) for each dataset in Table 4, and report the mean correlation for each category. Although high correlations with a gold standard dictionary may illustrate that the Grievance Dictionary is comparable to the LIWC in terms of psychometric qualities, we do not expect such a pattern because the Grievance Dictionary categories were designed to *supplement* LIWC categories and not replace them. Reported correlations serve to illustrate which other psycholinguistic concepts measured through the LIWC are related to each respective Grievance Dictionary category. The three highest correlating LIWC categories for each Grievance Dictionary category are depicted in Table 4 (full list of correlations available on OSF).

**Table 4:** Correlations (with confidence interval) Grievance Dictionary and LIWC

Category	Strongest correlating LIWC categories		
Deadline	cause: 0.10 [0.06–0.13]	drives: 0.06 [0.03–0.09]	work: 0.11 [0.06–0.16]
Desperation	discrep: 0.27 [0.15–0.40]	sad: 0.16 [0.08–0.25]	verb: 0.13 [0.09–0.16]
Fixation	insight: 0.24 [0.15–0.33]	pronoun: 0.18 [0.08–0.29]	verb: 0.20 [0.12–0.27]
Frustration	feel: 0.17 [0.07–0.26]	negemo: 0.13 [0.07–0.19]	sad: 0.09 [0.05–0.14]
God	affiliation: 0.21 [0.10–0.31]	posemo: 0.14 [0.11–0.18]	relig: 0.32 [0.12–0.52]
Grievance	affect: 0.08 [0.07–0.09]	negemo: 0.16 [0.06–0.26]	sad: 0.12 [0.05–0.18]
Hate	affect: 0.09 [0.06–0.12]	anger: 0.23 [0.12–0.34]	negemo: 0.15 [0.09–0.21]
Help	affect: 0.17 [0.10–0.25]	posemo: 0.20 [0.14–0.26]	reward: 0.23 [0.12–0.35]
Honor	affect: 0.18 [0.09–0.27]	drives: 0.16 [0.07–0.26]	posemo: 0.22 [0.12–0.32]
Impostor	power: – 0.03 [– 0.04–0.02]	relativ: – 0.05 [–0.09--0.02]	space: – 0.04 [–0.07–0.02]
Jealousy	cogproc: 0.11 [0.06–0.16]	discrep: 0.07 [0.05–0.10]	insight: 0.15 [0.07–0.23]
Loneliness	discrep: 0.06 [0.03–0.10]	sad: 0.08 [0.03–0.13]	time: 0.06 [0.04–0.08]
Murder	affect: 0.09 [0.04–0.13]	anger: 0.20 [0.10–0.31]	negemo: 0.17 [0.07–0.27]
Paranoia	anx: 0.11 [0.05–0.17]	cogproc: 0.08 [0.04–0.13]	negemo: 0.11 [0.06–0.16]
Planning	Authentic: 0.13 [0.05–0.21]	focuspresent: 0.14 [0.08–0.19]	insight: 0.15 [0.07–0.23]
Relation.	affiliation: 0.28 [0.12–0.43]	family: 0.23 [0.13–0.33]	social: 0.28 [0.10–0.46]
Soldier	achieve: 0.12 [0.10–0.15]	drives: 0.15 [0.12–0.18]	power: 0.17 [0.09–0.25]
Suicide	death: 0.16 [0.09–0.23]	health: 0.17 [0.07–0.28]	sad: 0.14 [0.08–0.21]
Surveillance	affect: – 0.05 [–0.07–0.02]	anger: – 0.04 [–0.06–0.02]	negemo: – 0.04 [– 0.06–0.02]
Threat	anger: 0.23 [0.13–0.33]	negemo: 0.17 [0.10–0.25]	Tone: – 0.14 [– 0.20–0.07]
Violence	anger: 0.21 [0.10–0.32]	death: 0.20 [0.09–0.32]	negemo: 0.28 [0.10–0.45]
Weaponry	negemo: 0.10 [0.05–0.15]	posemo: – 0.07 [–0.11–0.04]	Tone: – 0.11 [–0.16–0.05]

*Note.* All correlations were statistically significant at the *p* < 0.0023 (0.05/22 categories) level.

Overall, correlations were low (but statistically significant), suggesting that the Grievance Dictionary does not measure precisely the same constructs as the LIWC. Most Grievance Dictionary categories were correlated to LIWC categories which one might expect to be psychologically related. For example, several Grievance Dictionary categories such as frustration, grievance, hate, murder, paranoia, surveillance, violence, and weaponry were positively correlated to the LIWC category negative emotion. Hate, murder, surveillance, threat, and violence were also positively related to the LIWC’s anger category. These results may suggest that some LIWC categories serve as ‘umbrella categories’ for some in the Grievance Dictionary. That is, the LIWC can provide measures of more general concepts such as negative emotion, whereas the Grievance Dictionary is suited to give more granular measures of psychological constructs (e.g., frustration, paranoia) which fall into this overarching category.

### Part II: Dictionary validation

The dictionary validation reported in this section serves to assess whether and how the Grievance Dictionary can be used to distinguish between different types of writing, for example neutral language and grievance-fueled communications produced by terrorists or extremists. We first apply the Grievance Dictionary to different datasets to assess its external validity. Then, we test the performance of the dictionary in classification tasks.

#### External validity

We apply the dictionary to different datasets to test its validity in the context of grievance-fueled writings. All datasets are reported in Table 5. For the lone-actor terrorist sample, we draw sequential 100-word chunks from 22 manifestos resulting in a total sample of 4572 documents. This ‘chunking’ is performed so that the average word count for the terrorist manifestos is more comparable to that of the neutral writings and Stormfront posts. In order to demonstrate the extent of dictionary matches, Table 6 shows the mean percentage of word matches per dataset for each category. The last row of Table 6 also shows the mean proportion of words in the documents which were *not* matched with any word in the dictionary. These results show that most matches with the Grievance Dictionary were found in the lone-actor terrorist manifestos (44%) and the least matches were found in the in the neutral texts from blogs and forums (18%).

**Table 5:** Corpora used for statistical tests

Corpus	No. of documents	Mean word count (SD)
Lone-actor terrorist manifestos	4572	100 (4)
Neutral texts from blogs and forums	680,792	243 (503)
Stormfront posts	461,950	95 (229)
Stream-of-consciousness (SOC) essays	789	121 (35)
Abusive writing directed at politicians	789	121 (38)

**Table 6:** Mean dictionary matches (percentage) per dataset

Category	Lone-actormanifestos	Neutraltexts	Stormfrontposts	SOC	Abusivewriting
Deadline	2.58	1.47	1.23	2.38	1.57
Desperation	0.83	0.76	0.67	2.32	0.95
Fixation	0.38	0.71	0.58	2.04	1.02
Frustration	0.52	0.38	0.29	1.91	0.55
God	2.81	0.67	0.72	0.63	0.75
Grievance	0.56	0.38	0.40	1.71	0.56
Hate	2.04	0.50	0.84	1.83	1.29
Help	1.75	1.22	1.26	1.25	1.28
Honor	1.75	0.55	0.73	0.61	1.27
Impostor	0.34	0.15	0.20	0.09	0.44
Jealousy	0.55	0.29	0.29	1.35	0.46
Loneliness	0.79	0.96	0.84	1.87	1.03
Murder	3.22	0.87	1.30	0.96	1.45
Paranoia	0.82	0.49	0.45	1.93	0.56
Planning	3.68	1.81	1.72	2.89	2.04
Relationship	3.56	2.26	2.29	2.97	2.49
Soldier	4.17	0.70	1.22	1.05	1.01
Suicide	1.85	0.83	0.80	1.27	0.95
Surveillance	2.41	1.17	1.40	0.89	1.11
Threat	2.52	0.38	0.77	0.67	0.86
Violence	3.74	0.67	1.25	0.77	1.30
Weaponry	3.00	0.37	0.87	0.18	0.55
No match	56.14	82.44	79.89	68.43	76.52

In total, three statistical tests are performed on the proportional matches (category matches per document / total number of words per document) shown in Table 6. First, following previous work on violent language use (Kaati, Shrestha, & Cohen, 2016), we make statistical comparisons between the lone-actor terrorist manifestos and the neutral ‘control’ texts retrieved from online forums and blogs.^5^ Second, we perform a comparison between the lone-actor terrorist manifestos and the posts from right-wing extremist forum Stormfront.^6^ For both tests, mean dictionary outcome values of the lone-actor terrorist manifestos are compared to the means of the control samples with an independent samples *t* test. The control samples are down-sampled through bootstrapping to match the *n* of the lone-actor manifestos, with outcome measures reported as an average across 100 bootstrap iterations. We report the effect size for the difference by means of Cohen’s *d*^7^*,* in addition to the Bayes factor (BF). The Bayes factor is a measure of the degree to which the data are more likely to occur under the hypothesis that there is a difference in the dictionary categories between samples, compared to the hypothesis that there is no difference (Ortega & Navarrete, 2017; Wagenmakers, Lodewyckx, Kuriyal, & Grasman, 2010). For example, a BF between above 10 would constitute strong evidence for the alternative hypothesis that there is a difference (Ortega & Navarrete, 2017).

The third comparison is between abusive texts directed at politicians and neutral, stream-of-consciousness (SOC) essays (van der Vegt, Kleinberg, & Gill, 2020). For this comparison, a dependent samples *t* test is performed, because individual participants produced both types of text. Again, effect size *d* and BF are reported for the difference between the two samples (note that this comparison is not based on bootstrapping due to the smaller, equal sample sizes).

Results of each comparison are reported in Table 7. Overall, statistically significant differences were found for the majority of categories in all comparisons. In the majority of cases, the lone-actor manifestos scored higher on Grievance Dictionary categories than the control texts. In the first comparison with neutral texts from blogs and forums, the lone-actor manifestos scored higher on all categories except ‘fixation’ and ‘loneliness’ (denoted by a negative effect size *d*). The evidence for a difference between samples was very strong (BF > 10) in all cases except ‘desperation’. In the second comparison with Stormfront forum posts, the lone-actor manifestos scored proportionally higher on all categories except ‘fixation’ (strong evidence with BF > 10) and ‘loneliness’ (weak evidence BF < 10). For the comparison between abusive writing and stream-of-consciousness texts, differences in favor of SOC texts (BF > 10) were found (denoted by negative *d*) for the categories deadline, desperation, fixation, frustration, grievance, hate, jealousy, loneliness, paranoia, planning, relationship, and suicide. However, the abusive texts contained proportionally more references to honor, impostor, murder, violence, and weaponry (positive *d* and BF > 10).

**Table 7:** Statistical test results (effect size *d* with confidence interval and Bayes factor)

	Manifestos vs. neutral		Manifestos vs. Stormfront		Abuse vs. SOC	
	*d (bootstrapped)*	BF	*d (bootstrapped)*	BF	*d*	BF
Deadline	0.71 [0.70;0.71]	**531.5**	0.85 [0.85;0.86]	**759.93**	– 0.43 [– 0.52;– 0.31]	**62.75**
Desperation	0.07 [0.06;0.07]	1.69	0.16 [0.15;0.16]	**24.67**	– 0.88 [– 1.03;– 0.78]	**221.20**
Fixation	– 0.47 [– 0.48;– 0.47]	**243.56**	– 0.27 [– 0.28;– 0.27]	**78.67**	– 0.68 [– 0.82;– 0.57]	**146.28**
Frustration	0.21 [0.2;0.21]	**42.79**	0.34 [0.33;0.34]	**122.09**	– 0.87 [– 1.00;– 0.74]	**215.71**
God	0.87 [0.86;0.87]	**782.42**	0.84 [0.84;0.84]	**735.08**	0.10 [– 0.00;0.23]	0.85
Grievance	0.26 [0.26;0.26]	**74.05**	0.22 [0.21;0.22]	**50.00**	– 0.84 [– 0.97;– 0.73]	**205.73**
Hate	1.16 [1.16;1.17]	**>10**^**3**^	0.84 [0.84;0.84]	**735.28**	– 0.32 [– 0.43;– 0.20]	**34.72**
Help	0.41 [0.41;0.42]	**186.50**	0.36 [0.36;0.36]	**140.69**	0.03 [– 0.09;0.15]	-2.95
Honor	0.85 [0.85;0.86]	**765.27**	0.69 [0.69;0.70]	**513.27**	0.53 [0.41;0.65]	**91.99**
Impostor	0.36 [0.35;0.36]	**141.45**	0.23 [0.22;0.23]	**54.51**	0.45 [0.38;0.55]	**69.52**
Jealousy	0.38 [0.37;0.38]	**153.22**	0.36 [0.36;0.36]	**145.00**	– 0.72 [– 0.83;– 0.61]	**160.35**
Loneliness	– 0.17 [– 0.17;– 0.16]	**27.41**	– 0.05 [– 0.05;– 0.05]	– 0.29	– 0.57 [– 0.70;– 0.47]	**105.38**
Murder	1.27 [1.27;1.27]	**>10**^**3**^	0.96 [0.95;0.96]	**929.79**	0.33 [0.22;0.43]	**36.14**
Paranoia	0.39 [0.38;0.39]	**165.56**	0.42 [0.42;0.43]	**194.29**	– 0.99 [-1.11;– 0.88]	**263.82**
Planning	0.94 [0.94;0.95]	**915.80**	0.97 [0.97;0.98]	**968.30**	– 0.41 [– 0.53;– 0.29]	**56.93**
Relation.	0.55 [0.55;0.56]	**334.21**	0.52 [0.52;0.53]	**294.16**	– 0.21 [– 0.32;– 0.09]	**13.69**
Soldier	1.57 [1.57;1.57]	**>10**^**3**^	1.27 [1.26;1.27]	**>10**^**3**^	– 0.03 [– 0.14;0.07]	-2.82
Suicide	0.74 [0.74;0.75]	**581.54**	0.74 [0.74;0.75]	**585.71**	– 0.22 [– 0.34;– 0.12]	**15.80**
Surveillance	0.71 [0.70;0.71]	**531.55**	0.54 [0.54;0.55]	**326.47**	0.17 [0.05;0.27]	7.84
Threat	1.46 [1.46;1.46]	**>10**^**3**^	1.11 [1.10;1.11]	**>10**^**3**^	0.16 [0.05;0.27]	7.15
Violence	1.55 [1.55;1.55]	**>10**^**3**^	1.16 [1.16;1.16]	**>10**^**3**^	0.36 [0.26;0.49]	**44.94**
Weaponry	1.39 [1.39;1.40]	**>10**^**3**^	1.05 [1.05;1.06]	**>10**^**3**^	0.39 [0.30;0.48]	**51.38**

*Notes.* A positive *d* denotes a higher score on the category for the lone-actor terrorist manifestos (test 1 and 2) and abusive texts (test 3). A BF above 10 (in bold) constitutes strong evidence for the alternative hypothesis

#### Classification

Previous work classified terrorist or extremist texts from neutral ‘control samples’ using the LIWC. We investigate whether the Grievance Dictionary can achieve similar results, or increase prediction performance when used to supplement the LIWC.

##### Classification tasks

In three classification tasks, we examine whether the Grievance Dictionary and the LIWC can distinguish between:

1. Texts written by known terrorists vs. non-violent individuals
2. Texts written by known terrorists vs. non-violent extremists
3. Abusive vs. neutral texts (within-subject comparison of non-violent individuals)

All classification tasks were performed using a multinomial naïve Bayes classifier, a linear SVM, and a random forest model. We report the results for the best performing model. All analyses were performed in *R*, using the quanteda textmodels (Benoit et al., 2020) and randomForest (Liaw & Wiener, 2002) packages.

In Classification Task 1, we classify lone-actor terrorist manifesto excerpts (*N* = 4572) versus neutral posts from blogs and forums (*N* = 680,792). The majority class of neutral posts is down-sampled to the same *n* as the manifesto sample by means of bootstrapping (100 times), to allow for a balanced classification task. For each bootstrapped sample, we perform a five-fold cross validation using 80% of the sample as training data, and the remaining 20% as test data. Classification results are reported as an average across each of the five cross-validations across the 100 bootstrapped samples. In Classification Task 2, we classify lone-actor terrorist manifesto excerpts (*N* = 4572) versus Stormfront posts (*N* = 461,950). Following the same procedure as in Task 1, the majority class of Stormfront posts is down-sampled 100 times and cross-validated five times with an 80/20 split. In classification Task 3, we perform classification for abusive vs. neutral, stream-of-consciousness writing with data from van der Vegt et al. (2020), using 789 documents per sample. Note that due to the smaller sample size in Task 3 we do not perform bootstrapping, and instead opt only for a five-fold cross-validation with an 80/20 split.

##### Feature sets

Each classification task is performed using three different feature sets, to test the performance of the Grievance Dictionary, the LIWC and a combination of the two in classifying aforementioned datasets. The following feature sets are used:

- All 22 Grievance Dictionary categories.
- All psychological and social categories (*N =* 55) of the LIWC2015^8^. We exclude grammar categories from the LIWC such as pronouns and verbs because we are interested in the predictive ability of psychological concepts only, and grammatical categories do not appear in the Grievance Dictionary either.
- A combination of the Grievance Dictionary and psycho-social LIWC categories
  (*N* = 77).

##### Results of classification tasks

Performance metrics^9^ for the classification tasks are reported in Table 8. In all tasks, the random forest model performed best, with the exception of task 3b and 3c, where a linear SVM produced higher prediction performance. Classification Task 1 shows high performance for distinguishing between lone-actor terrorist texts and neutral texts. The Grievance Dictionary alone achieves 96% accuracy, which is further increased to 99% when using the LIWC. The combination of the LIWC and Grievance Dictionary does not provide a substantial improvement over the LIWC alone. Classification Task 2 similarly shows that the LIWC alone (and in combination with the Grievance Dictionary) achieves nearly perfect classification accuracy. In Task 3, the LIWC (alone and in combination with the Grievance Dictionary) similarly outperforms the Grievance Dictionary. Here, performance metrics are somewhat lower compared to Task 1 and 2, but the majority of cases are still accurately classified.

**Table 8:** Classification results

Task	Feature set	Accuracy	Kappa	Specificity	Precision	Recall
1. LA vs. neutral	a. Grievance	0.96	0.92	0.97	0.97	0.96
	b. LIWC	0.99	0.98	0.99	0.99	0.99
	c. Grievance + LIWC	0.99	0.98	0.99	0.99	0.99
2. LA vs. Stormfront	a. Grievance	0.94	0.87	0.94	0.94	0.94
	b. LIWC	0.99	0.98	0.99	0.99	0.99
	c. Grievance + LIWC^a^	0.99	0.99	0.99	0.99	0.99
3. Abuse vs. neutral	a. Grievance	0.83	0.67	0.86	0.86	0.82
	b. LIWC^a^	0.96	0.92	0.98	0.98	0.94
	c. Grievance + LIWC^a^	0.96	0.92	0.98	0.98	0.94

^a^The best performing model for these tasks was a linear SVM, rather than a random forest model (best performing in all other tasks)

##### Explaining high classification accuracies

All in all, classification accuracies were high, with several near ‘perfect’ performances. Therefore, we examined feature importance for each task in order to discover whether the model was biased towards some features. The five most important features for each task are reported in Table 9. Feature importance rankings are based on a ROC curve analysis, where a cut-off for each feature is defined that maximizes true positives predictions, and minimizes false positives; a larger area under the ROC curve implies larger variable importance (Kuhn, 2008). Tables with ROC values for each feature per task are available on the Open Science Framework.

**Table 9:** Feature importance per task (top five, full list of features on OSF)

Task	Feature set	Important features
1. LA vs. neutral	a. Grievance	soldier, weaponry, violence, impostor, threat
	b. LIWC	analytic language, present focus, power, differentiation, work
	c. Grievance + LIWC	analytic language, differentiation, present focus, soldier, violence
2. LA vs. Stormfront	a. Grievance	soldier, relationship, impostor, threat, hate
	b. LIWC	differentiation, analytic language, present focus, tentative, discrepancies
	c. Grievance + LIWC	differentiation, analytic language, present focus, tentative, discrepancies
3. Abuse vs. neutral	a. Grievance	paranoia, grievance, frustration, fixation, desperation
	b. LIWC	authentic language, social words, clout, feel, male
	c. Grievance + LIWC	authentic language, social words, clout, feel, male

Features with high importance also showed stark differences in mean proportional dictionary scores between datasets. For example, the most important feature ‘soldier’ in Task 1a showed a mean score for lone-actor terrorist manifestos of 0.04 (SD = 0.03), whereas neutral texts and Stormfront posts scored 0.01 (SD = 0.01) and 0.01 (SD = 0.01), respectively. This was reflected in the results observed in aforementioned Bayesian *t* tests, where a decisive difference (BF > 10^3^) was observed for ‘soldier’. The second most important feature ‘weaponry’ (BF > 10^3^) had a mean of 0.03 (SD = 0.03) in lone-actor manifestos, in contrast to 0.004 (SD = 0.01) and 0.01 (SD = 0.01) in neutral texts and Stormfront posts, respectively. These large differences between datasets will have contributed to the high prediction performance in this (and other) task(s), in that the classifier learned to over-rely on these features. In contrast, classification Task 3 showed somewhat lower performance compared to Task 1 and 2, likely because smaller differences between samples were observed. Indeed, the most important feature ‘paranoia’ scored 0.02 (SD = 0.01) in the stream-of-consciousness essays and 0.01 (SD = 0.01) in the abusive texts, with the Bayes Factor demonstrating a smaller difference (BF = 263.82) than the differences observed for the most important features in Task 1 and 2 (BF > 10^3^). Therefore, the model was perhaps less able to strongly rely on these feature differences. It remains to be seen in future research how the Grievance Dictionary performs on datasets with even smaller statistical differences between texts (e.g., violent texts written by individuals who want to actualize their threat, vs. similarly violent texts written by those who do not plan to actualize).

## General discussion

In this paper, we introduced the Grievance Dictionary, a psycholinguistic dictionary for grievance-fueled violence threat assessment. The aim of this work was to develop a dictionary which can specifically measure constructs relevant to threat assessment, and can be used for a wide variety of violence and extremism fueled by a grievance. Furthermore, we aimed to address the limitations we identified pertaining to existing psycholinguistic dictionaries. In this section, we examine the results obtained through statistical tests and classification tasks. This is followed by a discussion of the intended use for the Grievance Dictionary, as well as its limitations and possible future work.

### Linguistic differences

Based on the validation results of the dictionary, we saw that the Grievance Dictionary can elucidate differences between threatening and non-threatening language. Differences in Grievance Dictionary categories were found between texts written by lone-actor terrorists, neutral writing, and extremist forum posts, as well as between abusive language and stream-of-consciousness writing. The evidence for these differences was strong.

It must be noted that a high score on Grievance Dictionary categories is not exclusive to threatening and violent texts. In our comparison between stream-of-consciousness essays and abusive writing, the former obtained significantly higher scores for categories such as desperation, fixation, and frustration. Therefore, it is important to note that high scores on single dictionary categories should not be interpreted as individual risk factors for violence, as they may also occur in non-violent texts. Instead, the measures should be interpreted jointly to gain an understanding of the content of a grievance-fueled text, with particular attention paid to the highly ‘violent’ categories such as murder, violence, threats, and weaponry. Furthermore, the importance of Grievance Dictionary categories for distinguishing between different populations may also be context-dependent. For example, mentions of a (perceived) romantic relationship may positively predict violence in a threat directed at a public figure, while it may negatively predict violence (a ‘linguistic protective factor’) in an extremist text. Further research will be needed to establish and replicate differential meanings of Grievance Dictionary categories across contexts.

### Classification with the Grievance Dictionary

The dictionary categories were also used to classify different types of writing, including terrorist manifestos and extremist forum posts, neutral and extremist forum posts, as well as abusive and neutral writing. First, it is important to note that prediction was not the main objective for developing the Grievance Dictionary, because dictionary scores as features generally do not offer high prediction performance when compared to other features such as *n*-gram frequencies, parts-of-speech frequencies, or word embeddings (see e.g., Figea et al., 2016; Neuman, Assaf, Cohen, & Knoll, 2015; van der Vegt et al., 2020). However, since related research on extremism and terrorism has previously used the LIWC to classify text samples (Figea et al., 2016; Kaati, Shrestha, & Sardella, 2016), we found it important to examine whether the Grievance Dictionary can achieve the same. One benefit of using the Grievance Dictionary for prediction is that the contributing features remain interpretable to humans, in contrast to methods such as word embeddings which are difficult to interpret as features. Therefore, the Grievance Dictionary may be preferable in light of regulations such as the ALGOCARE framework, but it is important to realize that other more sophisticated (but less explainable) methods exist. The primary potential for the Grievance Dictionary thus does not lie within prediction, but in measurement (as demonstrated in the previous section on statistical differences). In this way, the Grievance Dictionary is more closely aligned to the risk assessment principles of Structured Professional Judgement than it is to actuarial approaches. The former is focused on understanding and formulating risk and linking this assessment directly to action. This process helps the user to consider the totality of circumstances that surround the individual being assessed. Actuarial approaches, on the other hand, are solely focused on prediction (Hart, Douglas, & Guy, 2016).

Nevertheless, the classification accuracy achieved in this study did approximate or outperform previous work in the violence research domain. The Grievance Dictionary alone already outperformed previous research, for example in classifying lone-actor terrorist manifestos from Stormfront posts (here: accuracy of 0.96 vs. 0.90 in Kaati et al., 2016). However, performance was further improved (sometimes to 99% accuracy) when using the LIWC (alone and in combination with the Grievance Dictionary). These results imply that although the Grievance Dictionary can achieve adequate prediction performance, it does not necessarily offer enhanced prediction performance over the LIWC. However, as has been raised previously, this was not the primary objective for developing the Grievance Dictionary. Moreover, the potential for obtaining more nuanced (violence-specific) measures with the Grievance Dictionary remains.

### Usage of the Grievance Dictionary

All things considered, the Grievance Dictionary shows promising results for demonstrating differences between different types of (non-)grievance-fueled language. Even though mean scores on dictionary categories were low (i.e., the majority of words across different datasets were not matched), values still elucidated strong differences between several (non) threatening texts. These results also suggest that the categories elicited from expert threat assessment practitioners hold value in understanding violent from non-violent language.

Perhaps, the most important academic use case for the Grievance Dictionary is to gain a general picture of language use in a (large) corpus, and to make (statistical) comparisons between different corpora. Because of the context-specificity of the dictionary, it may be especially suited to testing theories within the violence domain. Certain questions (e.g., Are right-wing extremists more paranoid than left-wing extremists? Do jihadists discuss weaponry more than right-wing extremists?) were previously not testable.

The Grievance Dictionary also shows promise for allowing practitioners to measure key concepts. This could help support practitioners to review all available written content in automated form and identify from within those data evidence for a range of features deemed relevant to the outcome to be prevented (e.g., a practitioner seeking to find those documents in a vast corpus that score high on weaponry). Alongside a consideration of the subjects’ other behaviors, it could help provide a basis for some form of summary judgement about risk, either its severity or imminence or a priority rating for the case, which is a recommendation of how soon action should be taken to mitigate the risks that appear to be posed (Douglas, Ogloff, & Hart, 2003). The Grievance Dictionary is intended as a decision-making tool towards risk management in the individual case rather than an actuarial method that indicates an individual’s similarity to a group of people with a known rate of offending. Thus, the Grievance Dictionary supports the consideration of a list of features, all of which have been derived through engagement with front-line practitioners.

Additionally, the Grievance Dictionary may also be used to gain a broad understanding of large-scale online social media data on a user or platform level, or to compare an incoming threatening message to a (police) database of existing communications. Furthermore, the tool opens up the possibility of studying grievance-fueled language in its full range, where Grievance Dictionary categories can be measured over time, for example to linguistically model processes of radicalization or extremism over time (e.g., Kleinberg, van der Vegt, & Gill, 2020) or in response to specific events (Burnap et al., 2014; van der Vegt, Mozes, et al., 2019; Zannettou, Finkelstein, Bradlyn, & Blackburn, 2019).

### Limitations and future work

In the current paper, we have endeavored to use the Grievance Dictionary to make meaningful comparisons between different types of violent and non-violent texts. Nevertheless, an important problem within the field of linguistic threat assessment persists. It is difficult to disentangle whether statistical differences emerged based on indicators for violence and non-violence or due to differences in topic. It is arguably not very difficult for the human eye or computer software to distinguish between a violent manifesto about attack planning and a blogpost about someone’s hobby. Of particular importance is performing linguistic comparisons between violent texts written by individuals who enact violent deeds, and the same amount of violent texts written by individuals not planning to act violently. If and when data from known violent individuals is more widely available, it will be of great interest to assess whether and how differences in Grievance Dictionary categories emerge, as well as how classification tasks perform. Another way to remedy this problem is with more experimental research, where both threat actualizers and bluffers produce texts (e.g., Geurts, Granhag, Ask, & Vrij, 2016) which can be assessed with the Grievance Dictionary.

Another limitation pertains to the construction of the dictionary. The seed words on which the dictionary categories are based were produced by human annotators who, to our knowledge, do not have violent ideations. Therefore, it may have been difficult for participants to produce words about attack planning and weaponry if they have little knowledge on the topic. We tried to somewhat ameliorate this problem by including word candidates obtained through automatic methods. Nevertheless, future improvements to the Grievance Dictionary may include word candidates that are obtained by means of a data-driven approach. That is, we may extract words from texts which are known to have been written by lone-actor terrorists or other violent individuals to serve as seed words.

A further limitation relates to the internal consistency of Grievance Dictionary categories. Although low internal consistency is generally expected for language-based measures (compared to self-report questionnaires, for example), the average reliability of Grievance Dictionary categories was lower than those observed for the LIWC (Pennebaker et al., 2015). This is somewhat surprising since LIWC categories were never intended to be semantically cohesive or comprehensive (Boyd & Schwartz, 2020), whereas our hope was to provide (somewhat) comprehensive linguistic measures of threat assessment concepts. These results potentially demonstrate the difficulty of cohesively measuring latent psychological concepts. Indeed, categories that can perhaps be considered as more abstract or difficult to interpret (grievance, fixation, impostor) scored lower on reliability than more concrete categories (soldier, weaponry), a factor dictionary users should also be aware of. It remains to be seen whether alternative (data-driven) wordlist generation procedures will result in higher internal consistency of categories.

Lastly, the assumption that the Grievance Dictionary categories indeed measure the (psychological) constructs they are designed to measure remains to be tested. For example, we do not yet know whether someone who is experiencing jealousy will also use more words from the jealousy category in the dictionary. This limitation holds for many psycholinguistic dictionaries including the LIWC, and highlights the importance of obtaining ground truth emotion datasets (Kleinberg, van der Vegt, & Mozes, 2020). Alternatively, emotions (and potentially other psychological constructs) can be experimentally manipulated prior to text writing in order to ascertain that the true emotional state of the text author is inferred from text (Kleinberg, 2020; Marcusson-Clavertz, Kjell, Persson, & Cardeña, 2019). Therefore, future work on the Grievance Dictionary and other psycholinguistic dictionaries should focus on measuring or even eliciting psychological processes such as frustration, jealousy, and loneliness, then measuring whether these constructs also emerge in language when applying the Grievance Dictionary.

## Conclusion

The purpose of the Grievance Dictionary is to serve as a resource for threat assessment practitioners and researchers aiming to gain a better understanding of grievance-fueled language use. Initial validation tests of the dictionary show that differences between violent and non-violent texts indeed can be detected using the dictionary. All information regarding the construction and specifications of the dictionary is available to researchers and practitioners, so that the capabilities *and* limitations of the Grievance Dictionary can be adequately scrutinized. Even though future research will be needed to ascertain the utility of the dictionary in other contexts (such as violent texts from authors with no violent intent), we hope the current work serves as an impetus to gain a better understanding of grievance-fueled language by automatic means.

## Footnotes

1 https://www.ibm.com/watson/services/tone-analyzer/

2 This includes endeavors such as PRAT (Akrami et al., 2018) and Threat Triage (Smith, Woyach, & O’toole, 2013)

3 As stated on the LIWC website: https://liwc.wpengine.com/

4 When using the weighted dictionary, users need to be aware that mean scores in the middle of the scale may be a result of disagreement (high standard deviations) between raters, rather than a reflection of ‘medium’ fit into a category (see Pollock, 2018).

5 The sample was drawn from the Blog Authorship Corpus (Schler, Koppel, Argamon, & Pennebaker, 2006) and the Boards.ie forum dataset from the 2008 SIOC Semantic Data Competition: https://semantic-web.com/2008/08/27/boardsie-sioc-semantic-data-competition-starts-september-1st/

6 All posts between 2012-2015 in the Stormfront dataset used in Kleinberg, van der Vegt, & Gill, (2020).

7 Cohen’s *d* expresses the magnitude of the difference after correcting for sample size. A *d* of 0.20, 0.50 and 0.80 can be interpreted as a small, moderate and large effect, respectively (Cohen, 1988)

8 Including the umbrella categories analytical thinking, clout, authentic language, emotional tone, affect words, social words, cognitive processes, perceptual processes, biological processes, core drives and needs, time orientation, relativity, personal concerns and informal speech (Pennebaker et al., 2015).

9 1) Classification accuracy: true positive + true negatives / true positives + false positives + true negatives + false negatives, 2) Kappa: observed accuracy – expected accuracy / 1 – expected accuracy, 3) Specificity: TN / TN + FP, 4) Precision: TP / TP + FP, 5) Recall: TP / TP + FN (see Sammut & Webb, 2011 for an overview).
